# Supplementary material for: Tead1a Initiates Transcriptional Priming Through the TEAD1a/YAP‐Notch1‐Spi1/Cebpα Axis to Promote Neutrophil Fate
Source: Adv Sci (Weinh). 2025 Aug 28;12(41):e05441. doi: 10.1002/advs.202505441 (PMC12591127; doi:10.1002/advs.202505441)
Supplement: Supplementary file 4 — Supplementary Dataset [file ADVS-12-e05441-s004.zip › 3-1.zip!/3-1/02-1.cluster/cluster0/Enrich/ego_all.pdf]

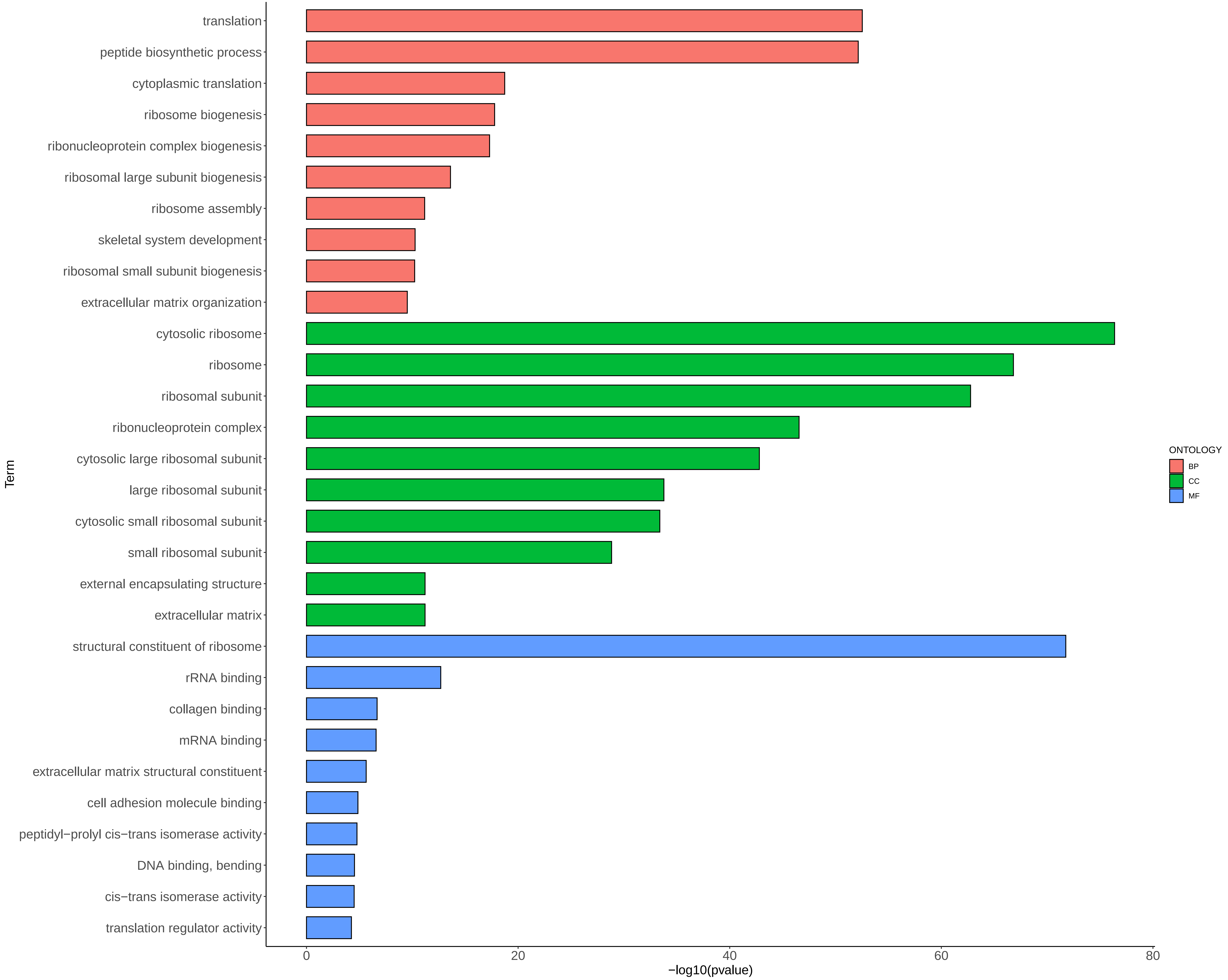
translation
peptide biosynthetic process
cytoplasmic translation
ribosome biogenesis
ribonucleoprotein complex biogenesis
ribosomal large subunit biogenesis
ribosome assembly
skeletal system development
ribosomal small subunit biogenesis
extracellular matrix organization
cytosolic ribosome
ribosome
ribosomal subunit
ribonucleoprotein complex
cytosolic large ribosomal subunit
large ribosomal subunit
cytosolic small ribosomal subunit
small ribosomal subunit
external encapsulating structure
extracellular matrix
structural constituent of ribosome
rRNA binding
collagen binding
mRNA binding
extracellular matrix structural constituent
cell adhesion molecule binding
peptidyl−prolyl cis−trans isomerase activity
DNA binding, bending
cis−trans isomerase activity
translation regulator activity
Term
0
20
40
60
80
−log10(pvalue)
ONTOLOGY
BP
CC
MF
